# How prepared is Mozambique to treat COVID-19 patients? A new approach for estimating oxygen service availability, oxygen treatment capacity, and population access to oxygen-ready treatment facilities

**DOI:** 10.1186/s12939-021-01403-8

**Published:** 2021-04-06

**Authors:** Langan Denhard, Parisa Kaviany, Sérgio Chicumbe, Cláudio Muianga, Guitunga Laisse, Kyle Aune, Ashley Sheffel

**Affiliations:** 1 Johns Hopkins Bloomberg School of Public Health, Baltimore, Maryland USA; 2 Johns Hopkins University School of Medicine, Baltimore, Maryland USA; 3 Programa de Sistemas de Saúde, Instituto Nacional de Saúde, Maputo, Mozambique; 4 World Health Organization, Maputo, Mozambique; 5 Hlayisa Project- Nweti, Maputo, Mozambique

**Keywords:** Geographic information systems, COVID-19, Oxygen, Preparedness, Mozambique, Modelling

## Abstract

**Background:**

This study aims to assess the COVID-19 response preparedness of the Mozambican health system by 1) determining the location of oxygen-ready public health facilities, 2) estimating the oxygen treatment capacity, and 3) determining the population coverage of oxygen-ready health facilities in Mozambique.

**Methods:**

This analysis utilizes information on the availability of oxygen sources and delivery apparatuses to determine if a health facility is ready to deliver oxygen therapy to patients in need, and estimates how many patients can be treated with continuous oxygen flow for a 7-day period based on the available oxygen equipment at health facilities. Using GIS mapping software, the study team modeled varying travel times to oxygen-ready facilities to estimate the proportion of the population with access to care.

**Results:**

0.4% of all health facilities in Mozambique are prepared to deliver oxygen therapy to patients, for a cumulative total of 283.9 to 406.0 patients-weeks given the existing national capacity, under varying assumptions including ability to divert oxygen from a single source to multiple patients. 35% of the population in Mozambique has adequate access within one-hour driving time of an oxygen-ready health facility. This varies widely by region; 89.1% of the population of Maputo City was captured by the one-hour driving time network, as compared ot 4.4% of the population of Niassa province.

**Conclusions:**

The Mozambican health system faces the dual challenges of under-resourced health facilities and low geographic accessibility to healthcare as it prepares to confront the COVID-19 pandemic. This analysis also illustrates the disparity between provinces in preparedness to deliver oxygen therapy to patient, with Cabo Delgado and Nampula being particularly under-resourced.

**Supplementary Information:**

The online version contains supplementary material available at 10.1186/s12939-021-01403-8.

## Background

The on-going COVID-19 pandemic has had a destabilizing effect on health systems, economies, and societies worldwide. Mozambique declared its first ever state of emergency on April 1, 2020, which was extended for the full 90-days allowable by the Constitution. In addition to mandating school closures, the order imposed strict limitations on workplaces, markets, public transportation, religious services, and leisure activities [1]. March 2020 models from the Imperial College of London, widely used by national governments in informing their COVID-19 responses, predicted devastation if the Mozambican government failed to act. The worst-case scenario predicts 65,000 deaths and 190,000 hospitalizations. Assuming a successful government response and suppression of social contacts by 75%, the model predicts 9000 total deaths and peak hospital demand of 19,000 [2].

Mozambique is a country in Africa located on the southeast coast, bordered by South Africa, eSwatini, Zambia, Zimbabwe, Tanzania, and Malawi. Mozambique gained independence from Portuguese colonial rule in 1975; Portuguese continues to be the only officially recognized language, though 43 unique languages are spoken throughout the country [3]. Its projected population is around 28 million people, over two-thirds of which live in rural areas. The country’s land area is 799,380 km^2^, and is divided into 11 provinces, including the capital, Maputo City [4]. The provinces in the northern region of the country are Niassa, Nampula, and Cabo Delgado. The central region is made up of Tete, Manica, Sofala, and Zambezia provinces, and the southern region consists of Inhambane, Gaza, Maputo, and Maputo City. The Ministry of Health’s Health Strategy for 2014–2019 identified the high prevalence of transmittable diseases including HIV, malaria, and tuberculosis to be a dominant factor in the shortened life expectancy at birth of Mozambicans. High levels of poverty, limited access to safe drinking water and quality health services, particularly in rural and northern regions of the country are primary drivers of the the heavy burden of disease in Mozamique. The health system primarily depends on external donors [5].

While the complete clinical picture of COVID-19 continues to evolve through rapidly published studies, it was first understood primarily through its impact on the respiratory system. The United States National Institute of Health (NIH) guideline currently recommends supplemental oxygen-therapy for patients with severe illness and ventilatory support for critically ill patients [6]. A study of treatment and clinical outcomes for a large cohort of over 72,000 patients in China found that 41.2% of hospitalized patients received oxygen therapy [7]. More recent data suggest that 15% of patients with COVID-19 develop severe symptoms requiring oxygen therapy, and 5% of all patients will require ventilation [8].

The COVID-19 pandemic and the threat of over-extended hospitals has prompted governments to assess their existing capacity against predicted demand. WHO guidance on COVID-19 preparedness advises that a health readiness assessment be conducted regardless of the current level of transmission and that steps be taken that all facilities, including first points of access to the health system, be able to provide basic emergency care to severe patients (WHO, Operational Considerations). A preliminary report on a 2018 assessment of health service availability and readiness, published by the National Institute of Health in Mozambique (INS) in February 2020 reported that nationally, there is an average of less than one (0.57) health facility and five inpatient beds per 10,000 people; Maputo City had the lowest number of health facilities per 10,000 people (0.33) but the highest ratio of inpatient beds (22.73 per 10,000). In contrast, Niassa had the highest ratio of health facilities per 10,000 people (1.03), and only 3.34 beds per 10,000 people. Comparatively, South Africa reported in 2010 having 23 beds per 10,000 people, Malawi reported 13 per 10,000 (2011), eSwatini reported 21 (2011, and Madagascar reported 2 (World Health Organization, *World Health Data Platform*) [9, 10]. Nationally, the country’s health facilities scored an average of 60% on the WHO global index of general readiness to provide basic health services [4]. The 2019 Global Health Security Index report found that no country was fully prepared for epidemic or pandemic response, based on performance on 34 indicators across six categories: (1) prevention, (2) detecting and reporting, (3) rapid response, (4) health system, (5) compliance with international norms, and (6) overall risk environment. The average index score across all countries was 40.2 out of 100; Mozambique ranked at 153 out of 195 countries with a score of 28.1 [11]. As a small number of rich countries secure the majority of the available supply of COVID-19 vaccine doses, poor and formerly colonized countries are faced with the prospect of living with the virus for years to come [12].

In addition to under-resourced facilities, geographic access to care has been studied as an additional barrier to care in Mozambique, most often in the context of measuring access to maternal health services [13–16]. There are also known limitations to modeling geographic accessibility using GIS software; it can be difficult to account for elevation, geographic barriers, and mixed methods of transportation. The “network analyst” model of using existing road networks and estimated rates of travel has widely replaced previous methods of modelling accessibility, such as straight-line Euclidian distance; this method has been shown to be more accurate and more practical for modelling [17]. A 2006 analysis of the reliabibility of modelled travel times found a strong association between travel times modelled by GIS software and time reported by patients [18]. A 2012 validation study found that the modelled travel times using a network analysis tool over-estimated observed ambulance travel times by 7–8 min [19]. Other studies conducted in Mozambique have demonstrated the need to model various travel scenarios to account for the mixed methods of traveling generally utilized by Mozambicans.

Mozambique has a total of 1651 public health facilities distributed throughout its 11 provinces [4]. A 2015 analysis of geographic accessibility to primary health clinics in Mozambique found significant geographic variability in coverage [13]. The authors modeled the number of villages within and beyond 60 min of a health care facility and found that 66.9% of Mozambique’s population lived within 60 min driving distance of a facility. In Maputo City, the proportion of population coverage increased to 100%. In the northernmost province, Cabo Delgado, the proportion of population covered fell to 48.2% [13]. A noted limitation of this study was that health facility capacity to offer specific services was not considered.

Geographic access to appropriately equipped health facilities is likely to be a barrier in providing care to patients requiring treatment for COVID-19 in rural areas. The aims of this study were to assess the COVID-19 preparedness of the Mozambican health system as a case study by 1) determining the location of oxygen-ready health facilities, 2) estimating the oxygen treatment capacity, and 3) determining the population coverage of oxygen-ready health facilities in Mozambique.

## Methods

### Data sources

#### Facility data

The Service Availability and Readiness Assessment (SARA) is a standardized health facility assessment tool developed by the WHO and used across low- and middle-income countries to assess and monitor health service availability and the readiness of facilities to deliver health-care interventions [20]. The tool assesses human resources, available beds, infrastructure and facility conditions, available services, and readiness to deliver services as measured by the availability of trained staff, guidelines, equipment, medicines and commodities, and diagnostics. GPS coordinates of all health facilities are also collected as part of the SARA survey. The Mozambique SARA was carried out by the Mozambique National Institute of Health (INS) in partnership with the Directorate of Planning and Cooperation at the Ministry of Health and with technical support from the World Health Organization. After adaptation to the country context and national health care standards in Mozambique, the SARA survey was implemented in a census of all public and private health facilities in Mozambique. The data were gathered by trained data collectors between April and August 2018. More details on the data collection process and procedures can be found in the Mozambique 2018 SARA report [4].

#### Spatial data

The following geospatial datasets were used in the analysis:

- Population distribution: A 30-m resolution population density raster was obtained from the Humanitarian Data Exchange (updated March 2020) (Facebook Connectivity Lab and Center for International Earth Science Information Network Columbia University, 2016)
- Administrative boundaries: Country, province, and district level shapefiles were obtained from the Humanitarian Data Exchange (updated June 2019)
- Road network: The road network was obtained from Open Street Maps (Open Street Map contributors, 2020). Additional fields were added for road class and type. Walking and driving times based on road class were added to the road network based on previous research in Mozambique [13]. (Supplementary Table 1)
- Health facility locations: GPS locations for all health facilities in the country were obtained from the 2018 Mozambique SARA dataset and converted into a shapefile.

All spatial files were projected into the ARC 1950/UTM 36S coordinate reference system to ensure the same projection across layers.

### Defining oxygen readiness

Criteria for oxygen readiness were developed by assessing the data available in the SARA questionnaire and through consultation with pulmonologists at the Johns Hopkins Hospital. Delivery of oxygen to a patient requires availability of an oxygen source and an oxygen delivery apparatus. Health facilities which had the functioning equipment available to administer oxygen via at least one of the following three options were considered “oxygen-ready”:

- Option A: Oxygen cylinder, flowmeter, and oxygen delivery apparatus
- Option B: Oxygen concentrator connected to power source and oxygen delivery apparatus
- Option C: Central oxygen supply connected to power source, flowmeter, and oxygen delivery apparatus

Detailed information on the specific survey questions utilized for each oxygen-ready option can be found in Supplementary Table 2. Additional equipment used for monitoring oxygen delivery include pulse oximeter probes and monitors to measure oxygen saturation levels of the patient and oxygen analyzers to check functionality of the levels of delivered oxygen (FiO2). However, these items remain uncommon in low- and middle-income countries, are not essential for delivering oxygen, and were not assessed in the SARA inventory. As such, for the purposes of this analysis, the availability of these items was not included in the definition of oxygen readiness. A facility was considered “ready” to deliver oxygen if it had all items required for at least one of the above oxygen readiness options.

### Determining oxygen treatment capacity

Oxygen treatment capacity was calculated to determine the number of patient-weeks for which a health facility could administer continuous oxygen flow, to facilitate comparison with projected single-week demand for care. Unlike oxygen concentrators and central oxygen supplies, the oxygen supply in cylinders is quite finite, and without information from the SARA survey on resupplies, empty cylinders are assumed to not be replaced or refilled. The SARA questionnaire collected information on the total number of cylinders, but did not ask about cylinder size. Using technical guidance from the World Health Organization, we provide information on common cylinder sizes in Supplementary Table 3 [21]. For the purposes of our analysis, we assumed that all cylinders were size J, with water capacity of 47 l and oxygen capacity of 6800 l, and that all cylinders were full at maxium capacity. The supplementary table provides the capacity of other standard cylinders and flow time at 5 and 10 l of oxygen per minute. Using the WHO technical specifications for oxygen sources, we assumed the pressure of the cylinder to be 1987 pounds per square inch (PSI) [8]. To calculate flow time, we assumed a maximum flow rate of 5 l of oxygen per minute for severely ill patients, due to the lack of information regarding facility access to a humidifier system.^1^ The formula to calculate flow time for cylinders is derived from Boyle’s Law, and provided as:

$$ t=\frac{P_r\times {V}_r}{P_i\times Q\times 60} $$

Where P refers to the pressure (PSI), and the subscripts “r” and “i” denote the remaining and initial cylinder pressure. V refers to volume (L), and Q refers to the flow rate (L/min). Multiplying the denominator by 60 converts the result from minutes to hours. The product, *t,* denotes the time in hours that the cylinder will last based on these conditions. Based on these assumptions and the above formula, a J-sized cylinder at full capacity could provide oxygen flow at a rate of 5 l per minute for 22.7 h. In order to provide one patient with continuous oxygen over the course of 7 days, the minimum number of J cylinders needed would be 7.4.

To determine capacity to deliver oxygen via concentrators, we assumed that one concentrator paired with one oxygen delivery apparatus could provide continuous oxygen over the course of 7 days. Oxygen concentrators function by taking in airfrom the environment using pressure swing absorption (PSA) technology and removing nitrogen to produce a continuous flow of oxygen. Concentrators are advantageous because they do not need to be continuously refilled, are portable, and can provide oxygen to multiple patients (CITE: WHO Oxygen Guidance). However, they rely on a consistent source of power. As such, health facilities that had concentrators as their sole oxygen source but did not have reliable power did not meet the criteria for oxygen-readiness. Each single concentrator and apparatus pairing was considered sufficient for one patient. To increase the sensitivity of our analysis, we also calculated a “high end” measure of capacity, assuming that health facilities were equipped with flowmeter stands, which can provide oxygen for up to 5 patients’ oxygen from a single concentrator, given sufficient number of oxygen delivery apparatuses.

To calculate capacity to deliver oxygen via a central oxygen supply, we assumed, based on the indicator criteria from the WHO SARA indicator reference guide and consultation with co-author PK, that one source of oxygen paired with one flowmeter and one oxygen delivery apparatus could provide continuous oxygen over the course of 7 days with a consistent source of power, and that the central oxygen supply could be routed to multiple patients simultaneously. Therefore, for facilities using a central oxygen supply, the capacity was limited by the number of flowmeter and delivery apparatus pairs.

### Facility treatment capacity

Several health facilities had multiple oxygen sources on hand, but insufficient equipment to deliver oxygen via multiple methods at once. For facilities that were equipped with cylinders in addition to concentrators and/or a central oxygen supply, we assumed that cylinders were available as backup but not as the primary source. If sufficient equipment were available to utilize multiple methods of delivery, the facility’s capacity to deliver oxygen via cylinder was calculated and added to the total. For example, to calculate the capacity of a hypothetical facility equipped with 3 concentrators, 2 cylinders, 1 flowmeter, and 3 delivery apparatuses, we assumed that the three delivery apparatuses would be used to supply oxygen from the concentrator for 3 patients continuously over 7 days, and the cylinders and flowmeter would not be used. In the event that this same facility actually had 4 delivery apparatuses, we would calculate the total weekly oxygen delivery capacity as 3 patients receiving oxygen via concentrator plus one patient that would receive oxygen via the two available cylinders and flowmeter for 22 h and 40 min; the total would therefore be 3.13 patients. We present these values as “patient-weeks” to facilitate interpretation.

### Descriptive statistics

We calculated descriptive statistics for key facility characteristics. Frequencies were calculated for oxygen-readiness by facility type and province. Among oxygen-ready facilities, the estimated number of oxygen treatment patient-weeks was calculated by province, facility type, and oxygen-delivery mode. The “low end” capacity range was calculated assuming that one concentrator paired with one oxygen delivery apparatus could provide continuous flow at a rate of 5 l per minute over the course of 7 days. Each single concentrator and apparatus pairing was considered sufficient for one patient. To increase the sensitivity of our analysis, we also calculated a “high end” measure of capacity, assuming that health facilities were equipped with flowmeter stands, which can provide up to 5 patients’ oxygen from a single concentrator, given a sufficient number of oxygen delivery apparatuses. All descriptive statistics were computed using STATA SE version 16 and Microsoft Excel.

### Geospatial analysis

Spatial analyses were conducted using QGIS version 3.10 and Arc Online. The QGIS network analyst tool was used to capture the road network within a half hour and hour of each oxygen-ready health facility, for driving and walking times. Three of the 57 oxygen-ready health facilities were not connected to the road network; road lines were added using Bing Imagery overlay in QGIS. The GPS coordinate data were missing for one oxygen ready health facility; these coordinates were incorporated by cross-referencing a list of health facility locations from the Humanitarian Data Exchange. A 500-m buffer was generated around each of the networks using Arc Online; the QGIS Zonal Statistics tool was used to extract the population raster statistics to the shapefile to capture the population within each of those networks. The Field Calculator was used to divide the population captured by the total population. The accessibility scenarios were then compared across provinces by clipping the dissolved network buffer to the extent of each province.

### Ethics

Permission to utilize the 2018 SARA data for this analysis was granted by the INS in Mozambique. This analysis did not involve human subject research.

## Results

### Facility characteristics

Of the 1651 public health facilities in Mozambique, 1643 were included in the survey. Eight health facilities in Cabo Delgado and Sofala were not included; areas of Cabo Delgado were impassable due to an on-going civil war, and areas of Sofala were geographically inaccessible to data collectors at the time of the study. The majority of health facilities (*N* = 1575) were rural and urban health centers (primary facilities), and 54 were district and rural hosptials (secondary facilties). Tertiary facilities include 7 provincial, military, and general hospitals. The remaining 7 facilities were operated at the quarterary level including the central hospitals in the major cities of Maputo, Beira, Nampula, and Quelimane, and 3 specialized hospitals. The majority of the facilities surveyed were in Nampula and Zambezia, the two most populous provinces. These two provinces also had the lowest proportions of facilities with consistent power (47.4 and 49.6%, respectively). Thirty-three of the 36 facilities in Maputo City had consistent power, as did 81.2% of the 122 facilities in Cabo Delgado. Each of the 4 provinces in the southern region had at least 1 inpatient bed per 1000 residents; this was only true for a single province (Sofala) outside of the south (Table 1).

**Table 1:** Facility characteristics, oxygen readiness, and oxygen delivery option available at oxygen ready facilities in Mozambique by province and facility level

		Inpatient Beds (per 1000 population)	Power available (%)	Oxygen-ready facilities (%)	Facilities surveyed (N)	Option A cylinders available (%)	Option B concentrators available (%)	Option C central supply available (%)	Oxygen-ready facilities (N)
**Region/Province**									
South/Maputo Province	663,817.1	2.4	66.1	5.4	112	83.3	66.7	50.0	6
South/Maputo City	2,101,098.4	1.3	91.7	19.4	36	85.7	85.7	57.1	7
South/Gaza	1,403,977.1	1.3	60.3	1.4	146	100.0	50.0	–	2
South/Inhambane	1,440,932.5	1.0	73.2	2.9	138	25.0	75.0	–	4
Central/Manica	1,928,572.2	0.6	70.0	1.7	120	100.0	50.0	50.0	2
Central/Sofala	2,023,637.8	1.1	51.0	0.6	157	–	100.0	–	1
Central/Tete	2,626,666.1	0.3	52.9	7.4	136	100.0	20.0	20.0	10
Central/Zambezia	5,017,685.6	0.5	49.6	2.8	254	85.7	71.4	14.3	7
North/Nampula	5,464,641.7	0.6	47.4	2.6	230	100.0	100.0	50.0	6
North/Niassa	1,742,122.0	0.6	63.0	1.0	192	–	100.0	–	2
North/Cabo Delgado	2,081,141.3	0.7	81.2	7.4	122	44.4	100.0	11.1	9
**Facility level**									
Primary			58.7	1.6	1575	60.0	60.0	4.0	25
Secondary			90.7	42.6	54	82.6	65.2	26.1	23
Tertiary			85.7	71.4	7	100.0	100.0	80.0	5
Quaternary			100.0	42.9	7	100.0	100.0	100.0	3
**National**		**0.8**	**60.0**	**3.4**	**1643**	**75.0**	**67.9**	**25.0**	**56**

### Oxygen-readiness

Fifty-six of 1643 health facilities (3.4%) were oxygen-ready, meaning they had at least one functioning oxygen delivery option available. The distribution of oxygen-ready facilities varied greatly across provinces. Of the 56 oxygen-ready facilities, most (10) were located in Tete province, followed by Cabo Delgado (9). Gaza, Niassa, and Manica provinces each contained 2 oxygen-ready facilties, while Sofala had 1 oxygen-ready facility.

The availability of oxygen-ready facilities was not proportional to the number of health facilities in a province. For example, Maputo City had 112 health facilities, representing 2.2% of the health facilities in the country, but the largest proportion of oxygen-ready facilities at 19.4%, accounting for 12.5% of the oxygen-ready facilities in the country. The next provinces with the largest proportion of oxygen-ready facilities were Tete (7.4%) and Cabo Delgado (7.4%). The province with the lowest proportion (0.6%) of oxygen-ready facilities was Sofala; however, Sofala had 157 health facilities, representing 9.6% of the health facilities and 1.8% of the oxygen-ready facilities in the country.

Oxygen-readiness also varied by facility type with more than two thirds (71.4%) of tertiary facilities being oxygen ready, while just under half of secondary and quaternary health facilities were oxygen-ready (42.6 and 42.9% respectively), and only 1.6% of primary facilities were oxygen-ready (Table 1).

All of the oxygen-ready facilities had an existing power source; 54 of them accessed a central electricity supply, 1 was equipped with solar panels, and 1 used another, unspecified source. 96.4% (*N* = 54) of the oxygen-ready facilities had a consistent and reliable electricity supply (Table 1).

The oxygen-delivery option available at oxygen-ready facilities varied with some facilities only having one oxygen-delivery option available while others had multiple oxygen-delivery options available. Just over half of oxygen-ready facilities (54%) had only 1 delivery option, 29% of oxygen-ready facilities had only 2 delivery options, and 20% of oxygen-ready facilities had all three delivery options available. The most commonly available oxygen delivery options were oxygen cylinders only (*n* = 15), oxygen concentrators only (n = 15), a combination of oxygen cylinders and concentrators (*n* = 13), and a combination of oxygen cylinders, concentrators, and central supply (*n* = 11). No facilities had only a central supply of oxygen or a combination of concentrators and a central supply of oxygen, while very few facilities (*n* = 3) had a combination of oxygen cylinders and a central supply (Table 2).

**Table 2:** Oxygen delivery options available at health facilities in Mozambique and oxygen treatment capacity by oxygen delivery option

Oxygen-Delivery Option^a^	Number of Health Facilities	Patient-weeks of oxygen treatment N (SD), low range	Patient-weeks of oxygen treatment N (SD), high range
Option A Only	15	8.4 (1.8)	8.4 (1.8)
Option B Only	15	20.0 (3.2)	52.0 (9.3)
Option C Only	0	–	–
Options A and B	13	37.5 (6.5)	99.6 (34.4)
Options A and C	3	79.0 (51.7)	79.0 (51.7)
Options B and C	0	–	–
Options A, B, and C	11	139.0 (42.2)	167.0 (54.5)

^a^Option A: Oxygen cylinder, flowmeter, and oxygen delivery apparatus

Option B: Oxygen concentrator connected to power source and oxygen delivery apparatus

Option C: Central oxygen supply connected to power source, flowmeter, and oxygen delivery apparatus

### Oxygen-treatment capacity

Nationally, the cumulative capacity of health facilities to supply continuous oxygen therapy at a rate of 5 l per minute for a period of 7 days in Mozambique ranged from 283.9 to 406.0 patient-weeks. Nationally, oxygen-ready facilities were able to provide 7-day continuous flow for an average of 5.1 patients-weeks per facility in the low range, and 7.2 patient-weeks in the high range scenario. The average number of patients that can be treated at a facility varied greatly across facility type and region. 21.4% of oxygen-ready facilities (*n* = 12), all of which were solely dependent on cylinders, were un-equipped to provide even one patient with continuous oxygen for a full week. Health facilities in Maputo Province, Maputo City, and Zambezia had the highest oxygen delivery capacity. There was high variability by province; 48.8–50.8 patients could receive oxygen delivery therapy continuously for seven days in Maputo City across 7 hospitals, whereas a single oxygen-ready hospital in Sofala could deliver oxygen therapy continuously to 1–5 patients (Table 3).

**Table 3:** Oxygen treatment capacity in Mozambique by province and facility level

	Number of Oxygen-Ready Health Facilities	Patient-weeks of oxygen treatment N (SD), low range	Average Patient-weeks per facility (SD), Low range	Patient-weeks of oxygen treatment N (SD), high range	Average Patient-weeks per facility (SD), High range
**Region/Province**					
South/Maputo Province	6	74.4 (57.2)	11.7 (23.7)	78.0 (56.6)	12.3 (23.5)
South/Maputo City	7	49.8 (18.9)	7.1 (7.1)	50.8 (18.6)	7.3 (7.0)
South/Gaza	2	1.7 (0.3)	13.5 (17.7)	1.7 (0.3)	27.0 (32.5)
South/Inhambane	4	4.0 (0)	2.1 (1.9)	11.0 (4.1)	3.1 (2.2)
Central/Manica	2	25.1 (24.9)	2.1 (2.7)	25.1 (24.9)	2.1 (2.7)
Central/Sofala	1	1.0 (−)	1.0(−)	5.0 (−)	5.0 (−)
Central/Tete	10	12.6 (5.0)	1.5 (1.7)	12.6 (5.0)	2.7 (4.8)
Central/Zambezia	7	52.2 (38.2)	7.0 (14.6)	60.2 (37.9)	8.2 (14.6)
North/Nampula	6	15.5 (3.6)	2.6 (1.5)	22.0 (4.8)	3.7 (2.0)
North/Niassa	2	3.0 (1.0)	1.5 (0.7)	11.0 (9.0)	5.5 (6.4)
North/Cabo Delgado	9	44.6 (24.6)	4.6 (8.0)	128.6 (51.5)	9.9 (12.3)
**Facility level**					
Primary	25	26.7 (4.6)	1.2 (1.0)	57.05 (13.5)	2.8 (3.8)
Secondary	23	135.2 (59.7)	5.8 (12.4)	202.9 (65.6)	8.3 (13.7)
Tertiary	5	62.0 (27.0)	12.1 (12.3)	86.0 (46.1)	16.9 (20.8)
Quaternary	3	60.0 (31.2)	20.0 (18.0)	60.0 (31.2)	20.0 (18.0)
**National**	**56**	**283.9 (78.1)**	**5.1 (10.4)**	**406.0 (91.9)**	**7.3 (12.3)**

There was also variation by facility level. Quartenary central and specialized hospitals could provide oxygen for an average of 20 patient-weeks per facility (in both the high and low range scenarios; these hospitals were more likely to use a centralized oxygen supply and were less impacted by the change in assumptions). Primary health centers could provide oxygen flow for an average of 1.2–2.8 patient-weeks per facility. Provincial, central, and specialized hospitals (secondary and tertiary facilties) made up 14.2% of the oxygen-ready facilities, but accounted for 43.0% of the total oxygen flow patient-weeks in the low-range scenario, and 36.0% in the high-range scenario. All of the quartenary facilities were equipped to administer oxygen via a central supply, compared to 4% of the primary health centers. All of the tertiary and quartenary facilities were equipped with oxygen concentrators as well, compared to 60.0% of primary facilities and 65.2% of secondary facilities.

### Geographic accessibility

**Fig. 1:**
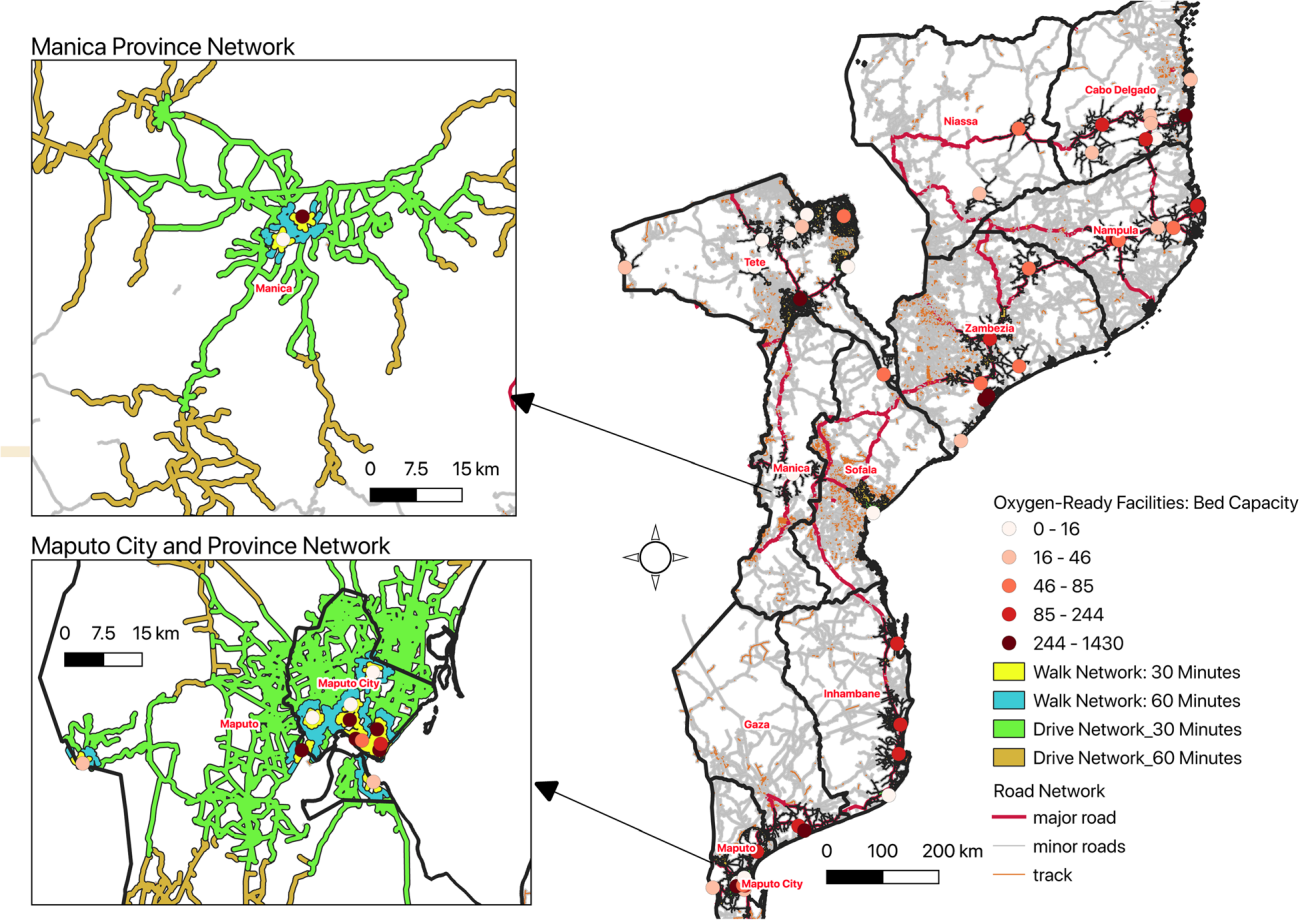
Network Analysis. Map of Mozambique displaying location of oxygen-ready facilities by bed capacity and modeled travel networks. Twocall-out boxes depicting portions of travel networks in Manica province (top left) and Maputo City and Province (bottom left)

Figure 1 depicts the geographic accessibility networks to oxygen-ready facilities in each of the four travel scenarios; each network includes the road travelled surrounded by a 500 m buffer. The differentiation between the walking and driving scenarios can be difficult to see on a large scale; the figure also zooms in on the networks surrounding the oxygen-ready facilities in Manica province and Maputo City. In Manica province, the networks, with the exception of the 30-min walking scenario, almost entirely overlap. In the South, travel networks straddled Gaza and Maputo provinces, and networks within Maputo City (zoomed-in segment, bottom left) stretched into the surrounding province. Many of the people living in Maputo province within an hour travel time to an oxygen-ready facility were, in fact, closest to a facility within Maputo City. Moving from the Northern to the Southern provinces, the map shows that the oxygen-ready facilties tend to be situated along the major national highway that runs through Mozambique.

Just over one-third (35.0%) of Mozambique’s total population was within 1 h driving distance of a health facility prepared to administer oxygen. That fell to 25.6% for the 30-min driving scenario. The population coverage of access to oxygen ready facilities in the walking scenario was dramatically lower. Nationally, 10.2% of the population lived within 60-min walking distance of an oxygen-ready facility; 5.5% of the population was captured in the 30-min walking scenario.

The accessibility of oxygen ready facilities varied greatly by province; within Maputo City, nearly 90% of the population lived within both 30 min and 1 h driving distance of an oxygen-ready facility (88.9 and 89.1% respectively). Niassa had the lowest proportion of population with access to an oxygen-ready facility (4.44%) in the 60-min driving scenario; Nampula (27.58%), Sofala (26.43%), and Manica (27.35%) also had low levels of population coverage. The proportion of the population living within an hour’s walking time of an oxygen-ready facility only rose above the national average (10.2%) in two provinces; Maputo City (50.3%) and Cabo Delgado (11.9%). These same two provinces were also the only two to reach above the national average (5%) population coverage in the 30-min walking scenario. There was a large variation in the proportion of the population with walking distance accessability to oxygen-ready facilities. 27.7% of Maputo City’s population lived within a half hour walking of an oxygen-ready facility, compared to 8.4% of Cabo Delgado’s population. The province with the third-largest proportion of coverage is Nampula (4.3%) (Table 4).

**Table 4:** Geographic accessibility to oxygen-ready facilities in Mozambique, by province

		% accessibility Driving Scenario		% accessibility Walking Scenario	
Region/Province	Total Population	60 min	30 min	60 min	30 min
South/Maputo Province	663,817.11	62.1%	49.8%	8.6%	3.6%
South/Maputo City	2,101,098.39	89.1%	88.9%	50.3%	27.7%
South/Gaza	1,403,977.07	33.5%	17.3%	5.1%	2.5%
South/Inhambane	1,440,932.50	40.9%	24.0%	7.0%	3.1%
Central/Manica	1,928,572.21	27.4%	19.0%	8.8%	3.1%
Central/Sofala	2,023,637.83	26.4%	21.5%	2.3%	0.5%
Central/Tete	2,626,666.06	51.6%	30.1%	6.7%	4.2%
Central/Zambezia	5,017,685.64	18.3%	11.9%	5.3%	3.1%
North/Nampula	5,464,641.65	27.6%	23.2%	8.6%	4.3%
North/Niassa	1,742,122.01	4.4%	3.6%	1.9%	1.7%
North/Cabo Delgado	2,081,141.31	47.6%	32.8%	11.9%	8.4%
**National**	**26,505,292**	**35.0%**	**25.6%**	**10.2%**	**5.5%**

## Discussion

This study assessed the COVID-19 preparedness of the Mozambican health system by determining the location of oxygen-ready public health facilities, estimating the oxygen treatment capacity, and determining the population coverage of oxygen-ready health facilities in Mozambique. Our analysis revealed that only 3.4% of health facilities in Mozambique are prepared to deliver oxygen therapy to patients in need and a total of 283.9 to 406.0 patients could be treated with continuous oxygen therapy for a week given the existing national capacity. In addition, only 35% of the population in Mozambique has adequate access within one-hour driving time of an oxygen-ready health facility. A 2015 study found that 66.9% of Mozambicans lived within an hour’s driving time from any primary healthcare facility, with the lowest levels of access found in Niassa and Zambezia and highest in Maputo Province and Maputo City [13]; the methodology used by the authors captured the entire population of a village that fell within the travel network. The findings of the INS’s analysis of the SARA census data showed that even for those who do live in close proximity to a health facility, many facilities lack the staff and equipment to offer basic, essential health services [4]. This analysis demonstrates the vulnerabilities of the Mozambican health system in the event of a widespread respiratory virus, related to both under-supplied health facilities and limitations in the transportation network.

This analysis also revealed major disparities in access to oxygen-ready health facilities across provinces and regions within Mozambique. In general, rural areas of the country not served by major highways lacked access to lifesaving treatment, while urban population centers had physical access to facilities but may also experience treatment shortages in case of a major disease outbreak. Southern provinces including Maputo Province, Maputo City, and Gaza contained the majority of oxygen-ready facilities and the most capacity to treat patients with oxygen therapy while provinces such as Sofala and Niassa had very little capacity. This regional disparity leaves the northern, largely rural population with limited access to care in the event of a widespread COVID outbreak. Resolving inequitable access to health care services is a recognized priority of the Mozambiquan government as evidenced by the most recent recommendations from Mozambique’s INS, which emphasized a focus on increasing the capacity of primary healthcare facilities, particularly in rural areas [4]. Existing disparities in health services will continue to be exacerbated by disruptions to the health system caused by a COVID outbreak and may further exacerbate differences in health outcomes observed in marginalized populations.

Despite the establishment of a large network of health facilities in every province, Mozambique has struggled to fulfill the government’s goal of universal health coverage and is unprepared for the new threat of the COVID-19 pandemic. Our findings have shown that the Mozambican health system faces the dual challenges of under-resourced health facilities and low geographic accessibility to healthcare. Primary health centers, which account for over 95% of all health facilities in Mozambique, are not simply the first point of access to healthcare for Mozambicans; they are often the only feasibly accessible option in rural areas. Our study found that while primary health centers accounted for the largest single share of oxygen-ready facilities (44.6%), they were only able to provide consistent oxygen flow for an average of 1.2–2.8 patient-weeks per facility. In March 2020, the Imperial College of London predicted that the peak hospital demand would reach 19,000 patients even if severe lockdown measures were imposed and enforced [2]. A more recent simulation by the Center for Disease Dynamics, Economics, & Policy predicted that there would be over 30,000 active severe infections on September 1, 2020 assuming lockdown measures and social distancing [22]. Assuming all infected patients had equal access to oxygen-ready facilities, our analysis found Mozambique would be able to provide a continuous oxygen flow to, at most, 2.1% of these patients. However, our analysis also found that there is not equal access to oxygen-ready facilities across provinces, leaving many parts of the country without access to care. This clearly demonstrates the lack of treatment capacity available nationally and highlights the need to focus efforts on expanding service capacity to underserved populations in order to mitigate the potential negative impacts of the COVID-19 pandemic in Mozambique.

This study has both limitations and strengths. The first, notable limitation of this analysis, which holds true for most GIS analyses focused on under-resourced countries, is the difficulty in simulating the mixed methods of transportation that may be utilized. However, we chose to generate a variety of travel scenarios, allowing us to detect patterns across travel scenarios. Furthermore, the road network shapefile was edited to reflect the road classes included in the network, such as paved or unpaved roads, and dirt trails, and we used existing literature to estimate travel times. There are inherent limitations in our data sources; the road network may not have captured all of the paths and tracks used. Furthermore, the accessibility of roads may vary across seasons; previous modeling found that flooding during the wet season significantly impacted the proportion of women of child-bearing age who live within one-hour walking time of a primary health center in the Southern region [14]. There are also limitations in the population raster, which calculates the national population to be 26,505,292 while the most recent census data count the population to be above 28 million. The health facility dataset excludes eight facilities, some due to geographic inaccessibility which was the focus of our study. In addition to measuring geographic accessibility, this study assessed the capacity of each equipped health facility to deliver oxygen over the period of one week. We were also limited by the nature of our dataset; the observed capacity captures a single point in time and did not incorporate the entirety of the supply chain. Oxygen cylinders are most dependent on a stable and reliable supply chain; were all oxygen cylinders to run out after this initial supply, that would only account for 8.4 patient-weeks of flow-time across 15 health facilities. It would be useful to have additional information on cylinder sizes and availability of flowmeter stands, and to have information on the frequency of supply re-stocks. The assumption we made regarding the size of the cylinder provided the most liberal estimate of oxygen delivery capacity; any smaller sizes would have reduced the flow time. Specific calculations can be made using the information provided on the varying standard cylinder sizes in supplementary Table 2. However, the vastness and recency of the SARA dataset makes it a useful tool for health facility capacity analyses and represents the most comprehensive information that can be used for planning outbreak response in Mozambique. The map produced includes information on the variation in bed capacity; this information as well as information on the number of clinicians and staff at each facility is included in the SARA dataset and could be the focus of further research. Furthermore, this analysis only included health facilities equipped with at least one of each of the accessories required for oxygen delivery. Other facilities surveyed in the census have one or more available components, but were missing essential equipment, such as delivery apparatuses, that could be potentially made quickly available. A further analysis may assess the costs associated with rapid scale-up. Information regarding supply chains and referral pathways would allow researchers and decision-makers to get a broader view of the health system’s capacity to confront COVID-19, beyond the patient-weeks of flow-time from the existing supply. Furthermore, insurgent violence in Cabo Delgado has intensified in recent months; as of December 18, 2020, UNHCR reported that an estimated 530,000 people were internally displaced in the northern region of the country and entire roads are impassable due to security concerns and poor conditions [23]. This may be reflected in the results of the study; as already stated, a small number of health facilities in Cabo Delgado were excluded from the SARA inventory for related reasons.

Finally, trends in transmission are likely to be influenced by emerging variants, as well as by the potentially mediating impact of vaccines. Variant 501Y.V2 was detected in South Africa and throughout Zambia in December 2020 and has been associated with increased transmissibility. Early data suggest that some vaccines may be less effective against this variant [24, 25]. While genome-sequencing has not be widely implemeneted in Mozambique, the variant has been detected in at least a dozen different countries including the United States [26], and may likely become the predominant strain in Mozambique which shares borders with both Zambia and South Africa. At the same time, global vaccine distribution will be inequitable. Mozambique has applied to receive vaccines through COVAX, a global resource-pooling system coordinated by WHO, the Coalition for Epidemic Preparedness Innovations (CEPI), and Gavi, the Vaccine Alliance. The health minister of Mozambique, Armindo Tiago, recently stated that the anticipated vaccine roll-out could begin in June or July, with vaccines to administer to 20% of their population by the end of 2021 [27]. This could be considered the “best case scenario.” Recent reports from COVAX add the strong caveat that this timeline is dependent on still uncertain funding availability and manufacturing capacity, as well as approval of secured vaccine candidates counted in the projected supply but still in development. Even if funding were to be obtained, the supply of vaccines able to be manufactured will be finite, and the vast majority have been pre-purchased by rich nations. A recent analysis by the Global Health Innovation Center at Duke University projected that it could be three or four years before the vaccine becomes widely available to people living in poor nations [28]. Storage and transportation requirements for approved vaccines are also anticipated challenges in many poor countries [29]. Without a clear timeline, projected vaccine supply, or developed infrastructure for vaccine distribution, is likely that the availability of supportive treatment for critically ill patients will remain a priority in Mozambique for the foreseeable future.

## Conclusion

The COVID-19 pandemic has spread to every populated continent and has strained health systems across the world. While the virus spreads easily across borders, it has exposed the structural inequalities ingrained in societies internationally, demonstrating that social determinants of health such as socioceconomic status and race are closely and unjustly correlated with one’s ability to access testing and treatment [30–32]. While donor-funded initiatives have prioritized the development of systems of response to HIV, malaria, and tuberculosis — certainly concerns of the Mozambican government as well— national efforts in building an infrastructure for universal health coverage have lagged and the health system is thus inadequately prepared to respond to a pandemic such as that caused by COVID-19. The results of this analysis can serve several purposes for both Mozambique and emergency response more broadly. Primarily and most immediately, it can be used to identify and prioritize vulnerable areas within Mozambique requiring additional support in the COVID-19 response, particularly the rural northern regions. Future research should examine the location-allocation of additional oxygen delivery resources in Mozambique to maximize the population able to receive care at an oxygen-ready facility. Importantly, this research models an approach for assessing vulnerability that can be used in other country contexts where the SARA census was conducted. Further research can incorporate additional indicators for preparedness and improve simulation of public transportation travel times. Finally, we hope it serves as a call to action for global health practitioners. As government agencies and external funders scramble to scale-up an adequate COVID-19 response, including resources for testing and treatment, we must acknowledge the failings and injustices that led to this moment of extreme vulnerability.

## Supplementary Information

**Additional file 1: Table S1.** Walking and driving times by road class. **Table S2.** Oxygen-ready detailed definitions. **Table S3.** Capacity of Oxygen Cylinders.

## Data Availability

The Service Availability and Readiness Assessment 2018 Mozambique dataset can be requested through this Google Form (hyperlinked here). After completing the form, email mozambiquesara2018@gmail.com. The Mozambique population raster used for this analysis is publicly available via Humanitarian Data Exchange (HDX). The country and province shapefiles are publicly available via the Humanitarian Data Exchange (HDX). The Mozambique Road Network was exported from Open Street Maps; this road network is continuously updated and is publicly available via the Humanitarian Data Exchange (HDX). Facebook Connectivity Lab and Center for International Earth Science Information Network Columbia University. 2016. Mozambique High Resolution Settlement Layer (HRSL). Updated 28 March 2020. https://data.humdata.org/dataset/highresolutionpopulationdensitymaps-moz. UN OCHA ROSEA (2019). Mozambique administrative level 0 (country), 1 (province), 2 (district) and 3 (posto) boundaries Updated 10 June 2019. https://data.humdata.org/dataset/mozambique-administrative-levels-0-3.

## Footnotes

1 A flow rate of 10-15 L/min is typically used for severly ill patients and would require a non-rebreather mask and a humidified system. As the SARA did not collect information on the availability of these items, we selected the default delivery apparatus (nasal cannula) for which it is not recommended to exceed 5 L/min using non-humified oxygen via nasal cannula.

## References

[1] U.S. Embassy in Mozambique: COVID-19 Information. 2020. https://mz.usembassy.gov/covid-19-information-2/. Accessed 13 May 2020.

[2] Walker PG, Whittaker C, Watson O, Baguelin M, Ainslie K, Bhatia S. The impact of COVID-19 and strategies for mitigation and suppression in low- and middle-income countries, WHO Collaborating Centre for Infectious Disease Modelling; MRC Centre for Global Infectious Disease Analysis; Abdul Latif Jameel Institute for Disease and Emergency Analytics. UK: Imperial College London; 2020.

[3] Lopes AJ (1998). The language situation in Mozambique. J Multiling Multicult Dev.

[4] Sabonete A, Momade A, Júnior A, Botão C, Baloi C, Muianga C, Ribeiro E, Amade E, Franco F, Muchanga G, Muianga J, Valido J, Matandalase M, Rambique O, da Costa P, Chicumbe S, Bignamini S, Gulamo Y (2020). SARA 2018 Inventário Internacional.

[5] Direcção de Planificação e Cooperação. Plano Estratégico do Sector de Saúde. República de Moçambique, Ministério de Saúde. September 30, 2013.

[6] National Institutes of Health: COVID-19 Treatment Guidelines. 2020. https://www.covid19treatmentguidelines.nih.gov/overview/. Accessed 13 May 2020. 34003615

[7] Wu Z, McGoogan JM. Characteristics of and important lessons from the coronavirus disease 2019 (COVID-19) outbreak in China: summary of a report of 72,314 cases from the Chinese Center for Disease Control and Prevention. JAMA. 2020;323(13):1239–42. 10.1001/jama.2020.2648 32091533

[8] WHO-UNICEF Oxygen sources and distribution for COVID-19 treatment centres; COVID-19 Clinical Care, Geneva: World Health Organization and United Nations Children's Fund (UNICEF); 2020.

[9] Yao J, Agadjanian V (2018). Bypassing health facilities in rural Mozambique: Spatial, institutional, and individual determinants. BMC Health Serv Res.

[10] World Health Organization. Hospital Beds (per 10,000 population). World Health Data Platform. http://www.who.int/data/gho/data/indicators/indicator-details/GHO/hospital-beds-(per-10-000-population. Accessed 9 Jan 2021.

[11] NTI and Johns Hopkins University Centre for Health Security (2019). Global Health Security Index.

[12] Twohey, M., Collins, K., & Thomas, K. 15, December 2020. With First Dibs on Vaccines, Rich Countries Have “Cleared the Shelves”. The New York Times. Accessed: January 10, 2021.

[13] Dos Anjos Luis A, Cabral P (2016). Geographic accessibility to primary healthcare centers in Mozambique. Int J Equity Health.

[14] Makanga PT, Schuurman N, Sacoor C, Boene HE, Vilanculo F, Vidler M, Magee L, Dadelszen P, Sevene E, Munguambe K, Firoz T (2017). Seasonal variation in geographical access to maternal health services in regions of southern Mozambique. Int J Health Geographics.

[15] Munguambe K, Boene H, Vidler M, Bique C, Sawchuck D, Firoz T, Makanga PT, Qureshi R, Macete E, Menéndez C, Von Dadelszen P, Sevene E (2016). Barriers and facilitators to health care seeking behaviours in pregnancy in rural communities of southern Mozambique. Reprod Health.

[16] Makacha L, Makanga PT, Dube YP, Bone J, Munguambe K, Katageri G, Sharma S, Vidler M, Sevene E, Ramadurg U, Charantimath U, Revankar A, Von Dadelszen P. Is the closest health facility the one used in pregnancy care-seeking? A cross-sectional comparative analysis of self-reported and modelled geographical access to maternal care in Mozambique, India and Pakistan. Int J Health Geographics. 2020;19(1). 10.1186/s12942-020-0197-5 PMC6998252 32013994

[17] Delamater PL, Messina JP, Shortridge AM, Grady SC. Measuring geographic access to health care: raster and network-based methods. Int J Health Geogr. 2012;15:11. 10.1186/1476-072X-11-15 PMC3511293 22587023

[18] Haynes R, Jones AP, Sauerzapf V, Zhao H. Validation of travel times to hospital estimated by GIS. Int J Health Geographics. 2006;5:40. 10.1186/1476-072X-5-40 PMC1586189 16984650

[19] Patel AB, Waters NM, Blanchard IE, Doig CJ, Ghali WA. A validation of ground ambulance pre-hospital times modeled using geographic information systems. Int J Health Geographics. 2012;11:42. 10.1186/1476-072X-11-42 PMC3527264 23033894

[20] O'Neill K, Sheffel A, Boerma T, Takane, Marina (2015). Service Availability and Readiness Assessment (SARA): An annual monitoring system for service delivery.

[21] WHO-UNICEF technical specifications and guidance for oxygen therapy devices; WHO medical device technical series; Geneva: World Health Organization and United Nations Children's Fund (UNICEF); 2019.

[22] Frost I, Osena G, Craig J, Hauck S, Gatalo O, Yang Y, Tseng K, Schueller E, Klien E, Lin G (2020). COVID-19 in East Africa: National Projections of exposed, Contagious, Symptomatic & Severe Cases.

[23] Baloch, Babar. More than 530,000 displaced in Mozambique’s conflict-torn north. 18, December 2020. UNHCR USA Accessed: January 9, 2021.

[24] Thomas, K., Zimmer, C., & LaFraniere, S. 28, January 2021. Novavax’s vaccine works well — except on variant first found in South Africa. The New York Times. Accessed: February 2, 2021.

[25] Zimmer, C., Weiland, N., & LaFraniere, S. 29, January 2021. Johnson & Johnson’s vaccine offers strong protection but fuels concern about variants. The New York Times. Accessed: February 2, 2021.

[26] Lauring AS, Hodcroft EB. Genetic variants of SARS-CoV-2—what do they mean? JAMA. 2021. 10.1001/jama.2020.27124. 10.1001/jama.2020.27124 33404586

[27] Reuters Staff. 13 January, 2021. Mozambique approaches global COVID-19 vaccine scheme for doses for 20% of population. Reuters. Accessed 1 Feb 2021.

[28] Duke Global Health Innovation Center. Launch and Scale Speedometer. Duke University. 2020. https://launchandscalefaster.org/covid-19. Accessed 2 Feb 2021.

[29] Figueroa J, Bottazzi M, Hotez P, Batista C, Ergonul O, Gilbert S, Gursel M, Hassanain M, Kim J, Lall B, Larson H, Naniche D, Sheahan T, Shoham S, Wilder-Smith A, Strub-Wourgaft N, Yadav P, Kang G. Urgent needs of low-income and middle-income countries for COVID-19 vaccines and therapeutics. Lancet. 2021. 10.1016/S0140-6736(21)00242-7. 10.1016/S0140-6736(21)00242-7 PMC7906712 33516284

[30] Brooks A. Was Africa rising? Narratives of development success and failure among the Mozambican middle class. Territory, Politics, Governance. 2018;6(4):447–67.

[31] Devi S. Cyclone Idai: 1 month later, devastation persists. Lancet. 2019;393(10181):1585. 10.1016/S0140-6736(19)30892-X. 10.1016/S0140-6736(19)30892-X 31007189

[32] Pfeiffer J, Chapman RR. NGOs, austerity, and universal health coverage in Mozambique. Global Health 15, 0. 2019. 10.1186/s12992-019-0520-8. 10.1186/s12992-019-0520-8 PMC6881911 31775785

