# Supplementary material for: How prepared is Mozambique to treat COVID-19 patients? A new approach for estimating oxygen service availability, oxygen treatment capacity, and population access to oxygen-ready treatment facilities
Source: Int J Equity Health. 2021 Apr 6;20:90. doi: 10.1186/s12939-021-01403-8 (PMC8022128; doi:10.1186/s12939-021-01403-8)
Supplement: Supplementary file 1 — Additional file 1: Table S1. Walking and driving times by road class. Table S2. Oxygen-ready detailed definitions. Table S3. Capacity of Oxygen Cylinders. [file 12939_2021_1403_MOESM1_ESM.docx]

***Supplementary Table 1: Walking and driving times by road class***

| **Road Class** | **Walking Travel Time** | **Driving Travel Time** |
| --- | --- | --- |
| **Primary** | 5 km/h (12 min/km) | 80 km/h (0.75 min/km) |
| **Secondary** | 4 km/h (15 min/km) | 50 km/h (1.2 min/km) |
| **Tertiary** | 4 km/h (15 min/km) | 20 km/h (3.0 min/km) |

***Supplementary Table 2: Oxygen-ready detailed definitions***

| ***Item*** | ***Category*** | ***Options*** | ***Indicator definition*** |
| --- | --- | --- | --- |
| Oxygen cylinder | Oxygen source | A | - Answered YES to “oxygen cylinders available?” - Answered YES to “oxygen cylinders functioning?” |
| Oxygen concentrator | Oxygen source | B | - Answered YES to “oxygen concentrators available?” - Answered YES to “oxygen concentrators functioning?” |
| Central oxygen supply | Oxygen source | C | - Answered YES to “central oxygen supply available?” - Answered YES to “central oxygen supply functioning?” |
| Oxygen delivery apparatus | Oxygen delivery apparatus | A, B, C | - Answered YES to “oxygen delivery apparatuses available?” - Answered YES to “oxygen delivery apparatuses functioning?” |
| Flowmeter | Additional accessories | A, C | - Answered YES to “Flowmeter available?” - Answered YES to “Flowmeter functioning?” |
| Power | Additional accessories | B, C | - Answered YES to “electricity available (any source)?” - Answered “All electrical needs of the facility” OR “Electrical lighting, communications, and 1 to 2 medical appliances” to “Electricity used for?” - Answered “Always available (no interruptions)” or “Often available some interruptions of less than 2 hours per day)” to “Electricity Reliability” |

***Supplemental Table 3: Capacity of Oxygen Cylinders***

| **Size** | **Oxygen Capacity (L)** | **Nominal Service Pressure** | **Flow Time (Hours) at rate of 5 L/min** | **Flow Time (Hours) at rate of 10 L/min** | **Standard Use** |
| --- | --- | --- | --- | --- | --- |
| **D** | 340 | 13 700 kPa (137 bar/1987 psi) | 1.3 | 0.6 | Emergency/Ambulance Transport |
| **E** | 680 | 13 700 kPa (137 bar/1987 psi) | 2.3 | 1.1 | Emergency/Ambulance Transport |
| **F** | 1360 | 13 700 kPa (137 bar/1987 psi) | 4.5 | 2.3 | Standalone |
| **G** | 3400 | 13 700 kPa (137 bar/1987 psi) | 11.3 | 5.7 | Standalone |
| **J** | 6800 | 13 700 kPa (137 bar/1987 psi) | 22.7 | 11.3 | Standalone and Manifold Connection |

Adapted from the WHO-UNICEF Technical Specifications and Guidance for Oxygen Therapy Devices: <https://apps.who.int/iris/bitstream/handle/10665/329874/9789241516914-eng.pdf?ua=1>
